# In vivo evaluation of a nanotechnology-based microshunt for filtering glaucoma surgery

**DOI:** 10.1038/s41598-024-54960-7

**Published:** 2024-02-23

**Authors:** Stefanie Gniesmer, Svenja Rebecca Sonntag, Anna Gapeeva, Ala Cojocaru, Sören Kaps, Rainer Adelung, Judith Sewing, Aysegül Tura, Salvatore Grisanti, Swaantje Grisanti

**Affiliations:** 1 Department of Ophthalmology, University of Luebeck, Luebeck, Germany; 2 Institute for Materials Science, Christian-Albrechts-University of Kiel, Kiel, Germany; 3 Phi-Stone AG, Kiel, Germany

**Keywords:** Glaucoma surgery, Microshunt, Microstent, Nanotechnology, ZnO-T, Subconjunctival space, Diseases, Eye diseases, Optic nerve diseases, Medical research, Preclinical research

## Abstract

To carry out the preclinical and histological evaluation of a novel nanotechnology-based microshunt for drainage glaucoma surgery. Twelve New Zealand White rabbits were implanted with a novel microshunt and followed up for 6 weeks. The new material composite consists of the silicone polydimethylsiloxane (PDMS) and tetrapodal Zinc Oxide (ZnO-T) nano-/microparticles. The microshunts were inserted ab externo to connect the subconjunctival space with the anterior chamber. Animals were euthanized after 2 and 6 weeks for histological evaluation. Ocular health and implant position were assessed at postoperative days 1, 3, 7 and twice a week thereafter by slit lamp biomicroscopy. Intraocular pressure (IOP) was measured using rebound tonometry. A good tolerability was observed in both short- and medium-term follow-up. Intraocular pressure was reduced following surgery but increased to preoperative levels after 2 weeks. No clinical or histological signs of inflammatory or toxic reactions were seen; the fibrotic encapsulation was barely noticeable after two weeks and very mild after six weeks. The new material composite PDMS/ZnO-T is well tolerated and the associated foreign body fibrotic reaction quite mild. The new microshunt reduces the IOP for 2 weeks. Further research will elucidate a tube-like shape to improve and prolong outflow performance and longer follow-up to exclude medium-term adverse effects.

## Introduction

The primary target to slow down the progression of glaucomatous optic nerve damage is still the intraocular pressure (IOP)^1^. When drug therapy, laser treatment or minimally invasive approaches fail, filtering surgery is required^2,3^.

Glaucoma filtration surgery is the most frequent and effective approach to reduce the IOP^4^. Despite an immediate effect, the long-term success however is often impaired by the postoperative wound healing responses^5,6^. Different drainage implants and different materials were therefore introduced to overcome this problem, but encapsulation of the implants is still a major complication, followed by IOP rise and leading to surgery failure^7–9^. It is known that inflammatory wound healing mechanisms caused both by surgery and the implant material and/or design are major determinants of success or failure^4,10^. Modern devices consisting of glutaraldehyde cross-linked porcine gelatin (XEN™, AbbVie, Wiesbaden, Germany) or Polystyrene-b-isobutylene-b-styrene (Preserflo™, Santen, Munich, Germany) have been suggested to reduce the fibrotic reaction. However, because of undeniable fibrotic responses, antimetabolites such as 5-fluorouracil (5-FU) and mitomycin C (MMC) as well as re-interventions are still required^11,12^.

The ultimate antifibrotic material has not been found yet, but nanotechnology may help to overcome the problem, that is primarily based on a foreign body reaction. In former studies we analyzed the antiproliferative effects of Zinc Oxide nano-/microparticles in a tetrapodal structure (ZnO-T) on primary cultures of human Tenon's fibroblasts (HTF). ZnO-T were able to reduce wound healing and also cytokine release and interfered with cellular growth on material consisting of ZnO-T combined with PDMS^13^.

Aim of this study was to obtain first in vivo results of biocompatibility, IOP reduction and histopathology of an innovative nanotechnology-based microshunt composed of PDMS and ZnO-T nano-/microparticles.

## Material and methods

All procedures conformed to the ARVO statement of the use of animals in ophthalmic research and our institutional guidelines. The study was approved by the local committee for animal use at the University of Luebeck (No. V 242-29443/2020 (111-10/19)). The study was reported in accordance with ARRIVE guidelines.

### Implant material

Implants were fabricated by the extrusion technique using polydimethylsiloxane (PDMS) and tetrapodal Zinc Oxide nano-/microparticles (ZnO-T). Medical grade ZnO-T nano-/microparticles were produced at Phi-Stone AG (Kiel, Germany). Medical grade PDMS MED-6820 (NuSil Technology LLC, Carpinteria, USA) was provided by HumanOptics AG (Erlangen, Germany)^14^.

To produce the stents, 75 wt% ZnO-T was mixed into the PDMS matrix. The stents were extruded from the polymer/particle mixture using a custom-made device. All components, except for nozzles, were made of stainless steel to withstand high pressure encountered during extrusion of the highly viscous polymer/particle mixture. Standard MK8 brass nozzles designed for 3D printers with bore diameters of 400 µm and 200 µm were used^14^.

**Figure 1:**
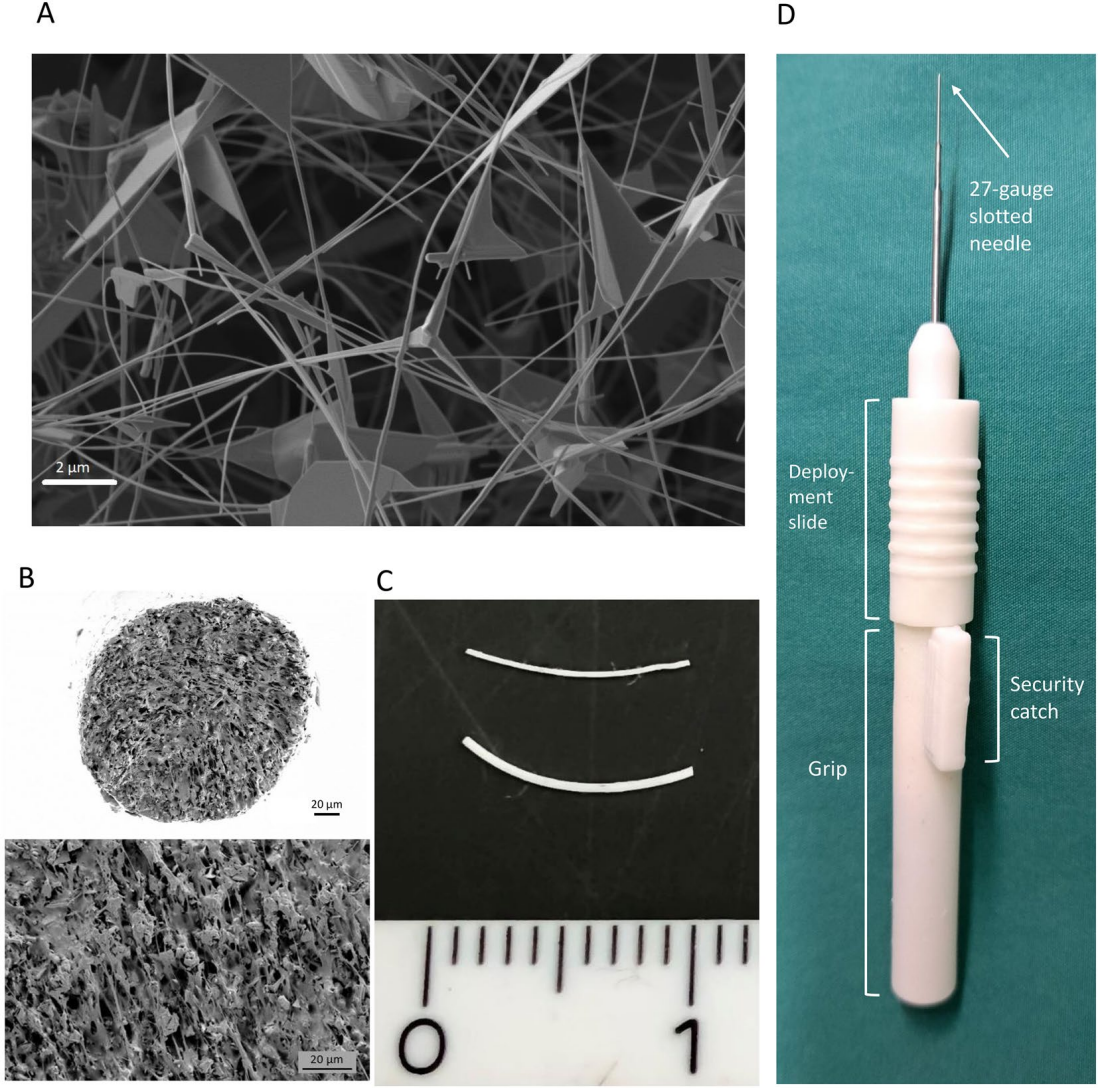
(**A**) Scanning electron microscope (SEM) shows the unique morphological structure of the tetrapods. (**B**) SEM micrographs of cross-sections of 400 µm stents at a magnification of: top 500×, bottom 1500×.(**C**) Photograph of the ready to use implants: top with 200 µm, bottom with 400 µm diameter. Length 10 mm. (**D**) Photograph of the inserter for the 200 µm implants.

The PDMS premixture was prepared by manually blending PDMS components A and B in a 1:1 ratio for a minimum of 5 min. Subsequently, ZnO-T particles were manually mixed into the PDMS premixture until a homogeneous powder-like blend was achieved. Following the extrusion process, the stents were suspended between two supports and left to dry in an atmospheric oven at 85 °C overnight. Once cured, the stents were cut to a length of 1.0 cm using a sharp blade^14^. Stents with 75 wt% ZnO-T and two different diameters (200 µm and 400 µm) were produced. The structure of ZnO-T nano-/microparticles is shown in Fig. 1A. The ready to use microstent is presented in Fig. 1B, C.

### Animals

All experiments were performed with female New Zealand White rabbits, 3 to 4 months old and weighing 1.5 to 2.5 kg. Animals were obtained from Charles River Laboratories (Sulzfeld, Germany) and acclimatized for at least 1 week before the experiments started. Prior to surgery, all animals were examined to exclude ocular diseases. Intraocular pressure was measured with a rebound tonometer (iCare Finland Oy, Vantaa, Finland). Twelve rabbits were subdivided into 4 groups (G1–G4) (Table 1). Each experimental group consisted of 3 animals. In the first set of experiments only a short follow-up of 2 weeks was planned. The two experimental groups (G1; G2) differed in the implanted size of the device (G1: 200 µm; G2: 400 µm). These two different sizes were also used for the second “medium-term” set of experiments with a follow-up of 6 weeks (G3: 200 µm; G4: 400 µm).

**Table 1:** Study groups.

Group	Description	Observation period (weeks)
G1	Implant with 200 µm outer diameter (n = 3)	2
G2	Implant with 400 µm outer diameter (n = 3)	2
G3	Implant with 200 µm outer diameter (n = 3)	6
G4	Implant with 400 µm outer diameter (n = 3)	6

### Surgical procedure

All rabbits (n = 12) received surgery on their right eye; the left eye was used as a control and received no surgical intervention. Animals were given an intramuscular injection of a ketamine-medetomidine mixture (35 mg/kg and 25 mg/kg, respectively). Additionally, all animals received topical anesthesia (Conjuncain EDO®, Bausch&Lomb, Berlin, Germany). All surgeries were performed by the same surgeon and assistant (Salvatore Grisanti and Stefanie Gniesmer) using an operating microscope (Zeiss OPMI, Jena, Germany). A 90° limbus-based conjunctival peritomy was made at 10 mm from the limbus in the superotemporal quadrant and the subconjunctival space was dissected anteriorly using Westcott scissors. Diathermy was not necessary. A scleral tunnel was created with a paracentesis starting from 1.5 mm posterior to the limbus and directed to the anterior chamber. A disposable inserter device with a grip, a 27-gauge slotted needle, and a deployment slide were developed (Fig. 1D) to facilitate insertion of the 200 µm sized implants. The 400 µm sized implants could be easily implanted using a non-teethed forceps without insertion device.

The 200 µm drainage implants were preloaded into the inserter (Fig. 1D). The preloaded inserter was then forwarded through the scleral tunnel. Once the implant was 3 mm in the anterior chamber, it was released from the inserter by retracting the inserter needle. The distal end was then placed under the conjunctiva. The 400 µm implants were easier to handle and placed in the same position by forceps. The conjunctiva was sutured with 10-0 Nylon in three cases at the opening site, because the implant appeared exposed. All implants were packaged sterile before being placed in the eye.

### Clinical evaluation

Clinical examination was performed by Slit lamp (Keeler ltd., Berkshire, United Kingdom) to evaluate the general appearance of the treated eyes, to assess inflammatory reactions, local toxicity and ocular intolerance, implant stability and to measure the IOP using the iCare® rebound tonometer (iCare™, Finland Oy, Vantaa, Finland). All examinations were performed without general or topical anesthesia on day 0 (baseline, before surgery) and day 1, 3, 7, 10, and 14 (group G1-G4), as well as day 17, 21, 24, 28, 31, 35, 38, and 42 (group G3-4). Additionally, the eyes were examined prior to scarification of the animals under general anesthesia. The IOP measurements were performed in triplicates to minimize measurement errors. All measurements were taken during the day. To exclude interindividual, cyclic and anesthesia-related variations, IOP was compared between the experimental right eye and the control left eye.

### Statistics

Statistical analysis of the IOP values was performed using SPSS 26 software (SPSS Inc., Chicago, USA). Data were tested by Shapiro–Wilk-Test for normality, and as they were not normally distributed, the Mann–Whitney-U-Test for independent samples was used to compare postoperative IOP between operated and non-operated eyes. α was 0.05 and levels of *p* < 0.05 were considered statistically significant.

### Histological evaluation

On postoperative day 14 (G1–G2) and 42 (G3–G4), animals were euthanized under general anesthesia with Pentobarbital 300 mg/kg body weight and the eyes enucleated together with the conjunctiva to preserve the bleb. The globes were immediately fixed in 10% formaldehyde for at least 24 h. Consequently, the eyes were examined, and a ring in the sagittal axis comprising the relevant area excised. Tissue samples were then dehydrated, embedded in paraffin, and cut as 5 µm serial sections.

For the hematoxylin–eosin staining, the sections were deparaffinized three times in xylene (J.T. Baker, Fisher Scientific, Schwerte, Germany), rehydrated in a descending series of 99%-50% ethanol (Roth, Karlsruhe, Germany) followed by distilled water for 5 min each, incubated in Meyer’s hematoxylin (Roth) for 7–10 min, and washed for 5 min under lukewarm tap water, followed by 2 rinses for 2 min in distilled water. The sections were then kept in 1% aqueous Erythrosin-B solution (Merck, Darmstadt, Germany), rinsed twice for 1 min each in distilled water, dehydrated in an ascending series of 50–100% ethanol, kept three times for 5 min in xylene, and mounted with Entellan (Merck).

For the Masson’s trichrome staining, the sections were deparaffinized and rehydrated as described above, followed by an incubation for 1 h at 56°C in Bouin’s solution (75 ml saturated picric acid (Merck), 25 ml of 37–40% formaldehyde (Th. Geyer, Renningen, Germany), 5 ml glacial acetic acid (Merck)) and an incubation for 10 min in Weigert’s hematoxylin (ROTH), with brief rinses under tap water and distilled water after every incubation. The sections were then kept in Biebrich scarlet-acid fuchsin solution (90 ml of 1% Biebrich scarlet, 10 ml of 1% acid fuchsin, 1 ml glacial acetic acid, all purchased from Merck) for 5 min, rinsed in distilled water, and incubated in a mixture of 2.5% phosphomolybdic acid—2.5% phosphotungstic acid (Roth) in distilled water for 15 min. The sections were stained with aniline blue (Chroma Gesellschaft, Köngen, Germany; 2.5 g aniline blue dissolved in 2 ml glacial acetic acid and 100 ml distilled water) for 2 min and rinsed in distilled water. After the incubation in 1% acetic acid (Roth) for 5 min, the sections were rehydrated as described above and mounted in Rotihistol (ROTH).

Light microscopic images were acquired via a color camera (Leica, Wetzlar, Germany, model DFC 295) that was attached to an inverted light microscope (Leica, model DMIL-LED) and by using the Leica Application Suite software (version 4.13.0, Leica).

## Results

### Slit lamp examination and clinical biocompatibility

**Figure 2:**
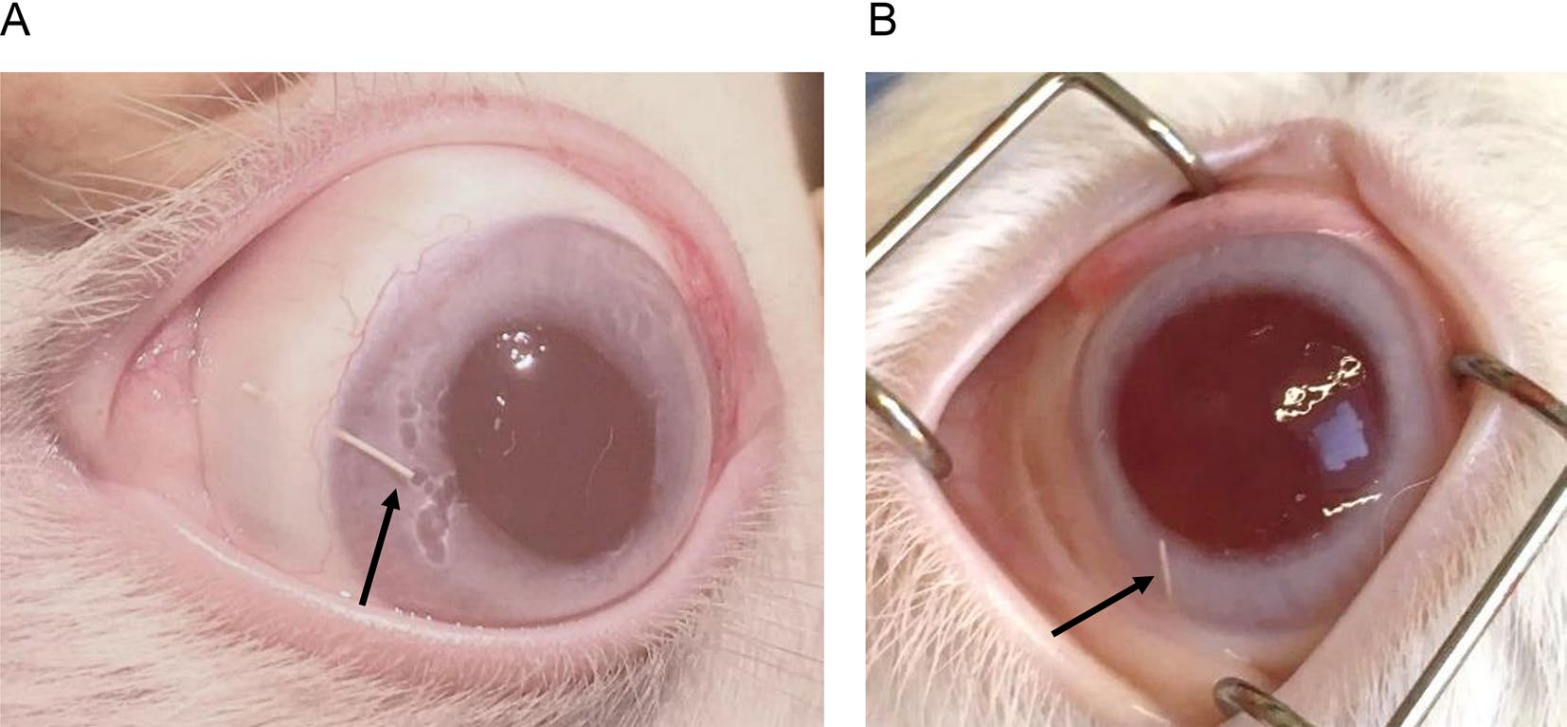
Clinical examination of the eyes two (**A**) and six (**B**) weeks after surgery, revealed, that the Microshunts (marked with black arrows) were in place without migration. No toxic changes nor inflammatory reactions were seen extra- and intraocular.

The postoperative phase was unremarkable in all 4 groups. None of the animals had signs of hypotony, hyphema or inflammation extra- or intraocular. The implants showed a stable position in the anterior chamber. Migration of the implant was not observed. The blebs were flat, but no external filtration was observed. At no time was there any shallowing of the anterior chamber, nor was there any hyphema. No signs of inflammation occurred, neither in the anterior chamber, nor at the implant site. Contact of the implant with the cornea or iris was not seen. There were also no signs of corneal toxicity (no swelling or opacification). The tissue surrounding the implants did not appear irritated. Due to the smooth and soft material of the microshunts, they adapted well to the curvature of the eyes. The implants therefore appeared to be well tolerated (Fig. 2).

### Tonometry

**Figure 3:**
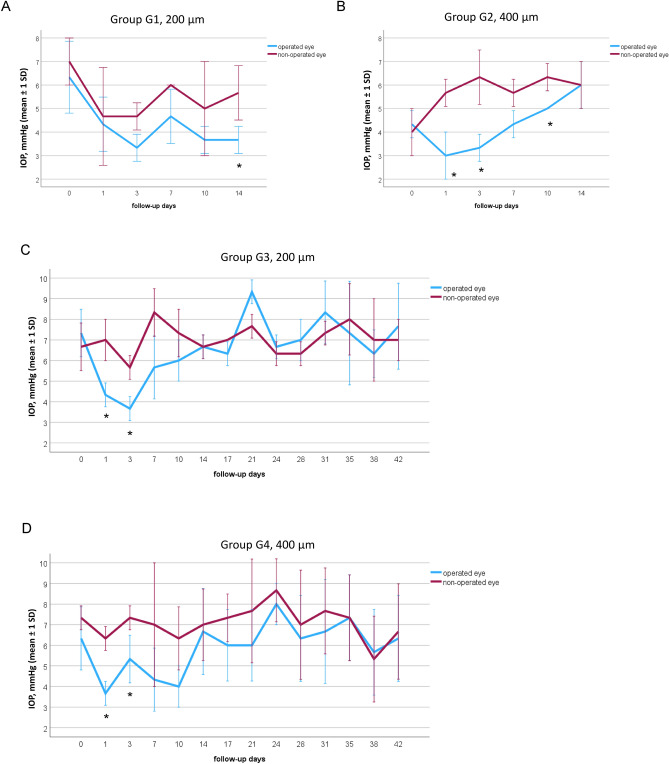
Intraocular pressure (IOP) of the operated right eye (blue) and the non-operated left eye (red), presented for the groups G1–G4. IOP was measured immediately before (day 0) as well as after surgery on days 1–14 for the short-term groups G1 (**A**) and G2 (**B**), and additionally on day 17–42 for the medium-term groups G3 (**C**) and G4 (**D**). (**A**) G1 implant with 200 µm outer diameter. (**B**) G2 implant with 400 µm outer diameter. (**C**) G3 implant with 200 µm outer diameter. (**D**) G4 implant with 400 µm outer diameter. Mann–Whitney-*U*-Test. Statistically significant values are marked with *.

In G1 (short-term, 200 µm diameter, Fig. 3A), the IOP was lower at all postoperative times in the operated eye than in the non-operated eye, but statistical significance was solely present on day 14 (p = 0.043, Fig. 3A, Table 2).

**Table 2:** Postoperative IOP measurements of groups G1–G4.

Group	IOP (mmHg)												
	Day 1	Day 3	Day 7	Day 10	Day 14	Day 17	Day 21	Day 24	Day 28	Day 31	Day 35	Day 38	Day 42
**G1 (200 µm)**													
Operated eye	4.33 (± 1.15)	3.33 (± 0.58)	4.67 (± 1.15)	3.67 (± 0.58)	3.67 (± 0.58)	–	–	–	–	–	–	–	–
Non-operated eye	4.70 (± 2.10)	4.70 (± 0.60)	6.0 (± 0.0)	5.0 (± 2.0)	5.67 (± 1.15)	–	–	–	–	–	–	–	–
**G2 (400 µm)**													
Operated eye	3.0 (± 1.0)	3.33 (± 0.58)	4.33 (± 0.58)	5.0 (± 0.0)	6.0 (± 1.0)	–	–	–	–	–	–	–	–
Non-operated eye	5.70 (± 0.60)	6.30 (± 1.20)	5.70 (± 0.60)	6.30 (± 0.60)	6.0 (± 1.0)	–	–	–	–	–	–	–	–
**G3 (200 µm)**													
Operated eye	4.33 (± 0.58)	3.67 (± 0.58)	5.67 (± 1.53)	6.0 (± 1.0)	6,67 (± 0.58)	6.33 (± 0.58)	9.33 (± 0.58)	6.67 (± 0.58)	7.0 (± 1.0)	8.33 (± 1.53)	7.33 (± 2.52)	6.33 (± 1.15)	7.0 (± 2.65)
Non-operated eye	7.0 (± 1.0)	5.70 (± 0.60)	8.30 (± 1.20)	7.30 (± 1.20)	6,67 (± 0.58)	7.0 (± 0.0)	7.7 (± 0.60)	6.33 (± 0.58)	6.33 (± 0.58)	7.33 (± 0.58)	8.0 (± 1.73)	7.0 (± 2.0)	7.0 (± 1.0)
**G4 (400 µm)**													
Operated eye	3.67 (± 0.58)	5.33 (± 1.15)	4.33 (± 1.53)	4.0 (± 1.0)	6.67 (± 2.08)	6.0 (± 1.73)	6.0 (± 1.73)	8.0 (± 1.0)	6.33 (± 2.08)	6.67 (± 2.52)	7.33 (± 2.08)	5.66 (± 2.08)	6.33 (± 2.08)
Non-operated eye	6.3 (± 0.6)	7.0 (± 1.0)	7.0 (± 3.0)	6.30 (± 1.50)	7.0 (± 1.73)	7.33 (± 1.15)	7.7 (± 2.5)	8.67 (± 1.53)	7.0 (± 2.65)	7.67 (± 2.08)	7.33 (± 2.08)	5.33 (± 2.08)	6.67 (± 2.31)

In G2 (short-term, 400 µm diameter, Fig. 3B), up to day 10, all IOP values of the operated eye were lower than those of the non-operated eye (Table 2, Fig. 3B). This IOP-reduction was significant on day 1 (*p* = 0.046), 3 (*p* = 0.043) and 10 (*p* = 0.034, Fig. 3B). On day 14 no IOP reduction could be seen.

In G3 (medium-term, 200 µm diameter, Fig. 3C), IOPs were lower on the operated eye up to day 10 (Table 2). The IOP reduction was statistically significant on day 1 (*p* = 0.046) and day 3 (*p* = 0.043). After 2 weeks, IOP increased to preoperative levels (Fig. 3C and Table 2).

In G4 (medium-term, 400 µm diameter, Fig. 3D), there was a similar trend of the IOP values as in G3 (Fig. 3D). A significantly lower IOP was observed on day 1 and day 3 (both *p* = 0.043). Thereafter the IOP increased and was at the preoperative level from day 14 (Fig. 3D, Table 2). Compared to the control eye, the IOP of the operated eye was still lower up to day 31. However, this was no longer significant (Fig. 3D).

### Histological results

**Figure 4:**
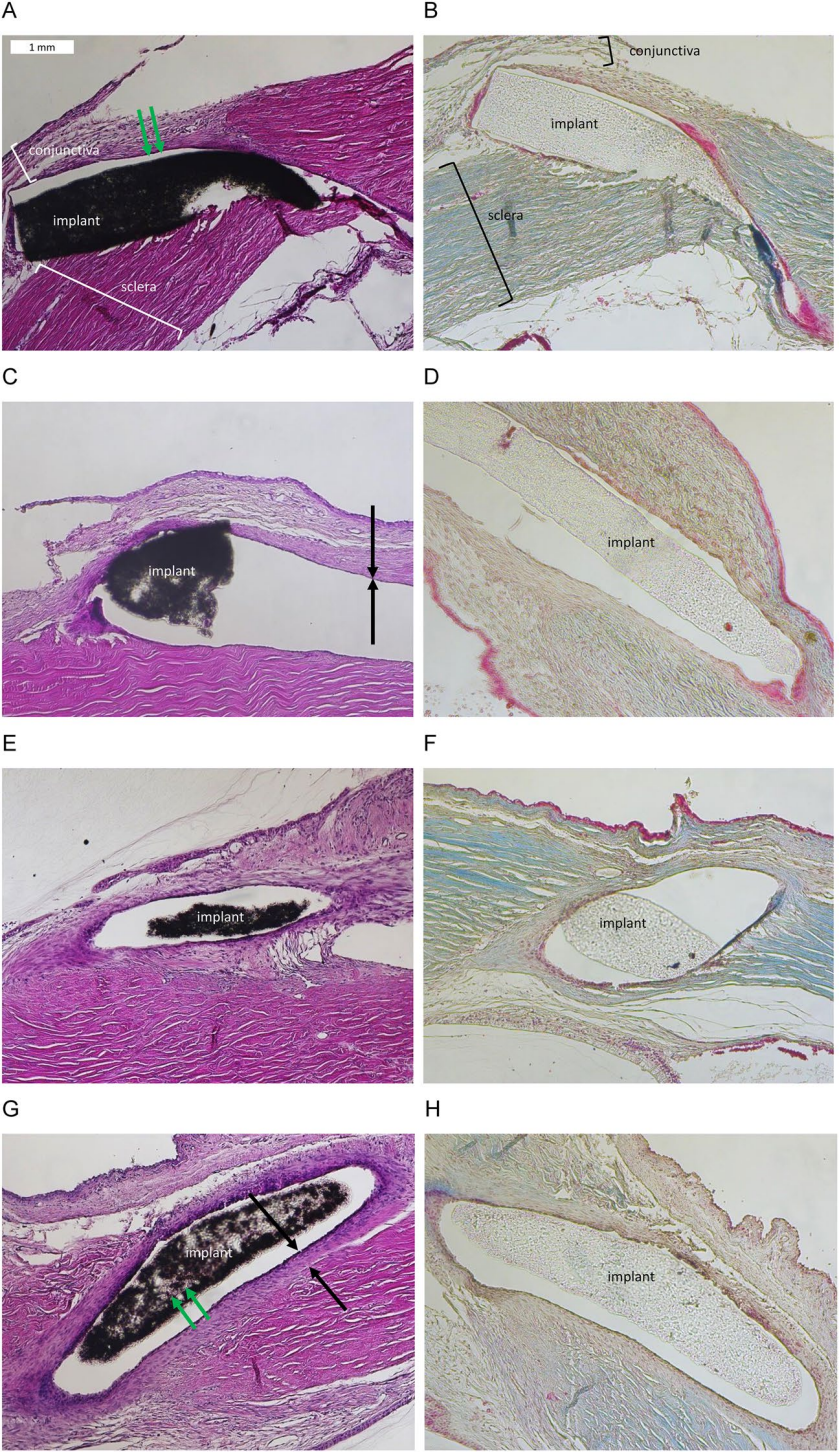
Microphotographs show the implant in its canal and the surrounding conjunctiva and sclera in different sections. Left column Hematoxylin / Eosin (HE) staining, right column Masson staining. In the HE stain, the implant is colored black and in the Masson stain, the implant is greyish. (**A**–**D**) after two weeks, microphotographs showed no cells at the surface of the implant (marked with green arrows). A few cells were seen on the canal wall (marked with black arrows). (**E**–**H**) after 6 weeks, HE microphotographs showed more surrounding cells on the canal wall according to a discrete encapsulation (marked with black arrows). Besides, on the surface of the implants a few cells were seen (marked with green arrows).

After two (G1 and G2) and six (G3 and G4) weeks, various histological sections along the microshunt were prepared and examined in Hematoxylin–Eosin (HE) and Masson staining as described above (Fig. 4).

After two weeks there was only a very mild cell reaction. Few cells were seen in the surrounding of the microshunt, but they did not form a circular fibrotic capsule (Fig. 4A–D). The implant itself showed no cell colonization (Fig. 4A–D). In particular, there was no evidence of excessive collagen deposition in both stainings. Furthermore, there were no signs of infiltration with inflammatory cells or toxic changes such as necrotic or disrupted cell morphology in sensitive structures such as the corneal endothelium or the retina (not shown).

After 6 weeks, few cells could be observed on the implant surface. In addition, more cells were found surrounding the implant, but still no continuous capsule was present (Fig. 4E–H). In all sections examined, collagen fibers could be seen at the implant-surrounding area. However, there was no evidence of inflammation or toxic changes in any of the eyes, with a normal, well-preserved morphology of the adjacent tissue. Furthermore, we did not observe any degradation of the implant and no abnormalities in the corneal endothelium.

## Discussion

Our previous studies showed promising results in reducing wound healing by ZnO-T in vitro^13,17^. Herein we can demonstrate that this innovative composite material made of PDMS and ZnO-T has a good biocompatibility with no clinical or microscopic detectable cytotoxic effects, without an inflammatory tissue reaction and with only a very mild cellular foreign body response. The tissue-implant interface on postoperative day 42 disclosed a thin cellular layer, but no fibrotic reaction as known from other studies in a rabbit model^10^. Nakamura et al. compared the tissue response after subconjunctival implantation of disks of 4 devices made of different materials: SIBS (Preserflo™), silicone (large-plate glaucoma implants), stainless steel (EX-PRESS®), and GACLC (XEN™). After 12 weeks, silicone showed the lowest tissue reaction^18^. This is in line with our results as our stent is partially made of the silicone polydimethylsiloxane (PDMS), and contradicts the work of Acosta et al. who reported less surrounding tissue reaction with SIBS tubes compared to silicone tubes. Van Mechelen et al., who implanted the Preserflo™ stent in rabbit eyes, found that the bleb failed 2 weeks postoperatively at the latest due to fibrotic responses^11^. In contrast, Fujimoto et al. found only minimal scarring after Preserflo implantation and observed functioning blebs even after 12 weeks ^16^. The morphology of the blebs in this study was flat, but without any external leakage. The suspected reason for this, is the slower flow with our implant compared to tube-like implants. In preliminary tests, wider stents with 6 mm in diameter were made from ZnO-T and PDMS using a different manufacturing process^17^. They were tightly sealed in a silicone tube and a certain water pressure was applied to the stents using compressed air and a pressure gauge. Starting at a pressure difference of 100 mbar, a low flow rate of up to 0.1 µl/min was observed (not shown). These flow measurements are not possible on stents shown in this study due to their small diameter, as they are manufactured directly in final dimensions (200 µm or 400 µm in diameter) using the extrusion technology^14^. The fact, that the IOP was postoperatively reduced, indicates the presence of outflow at the implant site. Due to the small diameter, the aqueous humour is supposed to disseminate slowly in the subconjunctival space to be steadily absorbed, which explains the flat bleb.

The rabbit filtering surgery model is known for its overwhelming fibrous reaction. In fact, other groups using the same model had no significant IOP reduction at all. In the study from Acosta et al., a poly(styrene-b-isobutylene-b-styrene) SIBS stent (Preserflo™) was compared to a silicone stent. With both stents, no significant decrease in IOP was found within 1 week^19^. In another study, van Mechelen et al. also found no significant IOP reduction after Preserflo™ implantation in rabbits compared to their non-operated eye^11^. However, a third study by Fujimoto et al. could show IOP lowering up to 12 weeks using the same stent^16^. A comparison of the three studies shows only minor differences in the surgical procedure.

Due to the minor depth of the anterior chamber in the rabbit model, we implanted our stent ab externo. Other available stents such as the XEN^TM^45 and XEN^TM^63, on the other hand, are implanted ab interno^20,21^. Shute et al. used the ab interno approach of XEN implantation in dogs but, in contrast to our study, found no significant IOP reduction^22^. An ab externo approach causes surgical trauma to the conjunctiva, which is minimized with the ab interno approach^20^. However, a retrospective study comparing Preserflo™ and XEN^TM^45 found comparable IOP reductions and surgical success for both stents in clinical use^23^. Furthermore, a growing number of surgeons implant the XEN Stent ab externo, because they find less bleb encapsulation than for the internal approach^24^.

In our study, we tested stents with two different outer diameters: 200 µm and 400 µm. The reason therefore was to verify (a) the feasibility of implantation of this material and (b) the volume and amount of material that might induce an inflammatory or toxic reaction.

The lumenless design stands in contrast to other subconjunctival stents, such as XEN^TM^45 or Preserflo™, but compares to the supraciliary implanted Miniject®. This latter microshunt consists of a soft, spongy material made of silicone. As far as it is known, it does not contain any other component, that suppresses a foreign body reaction. This might be the reason, why the Miniject® was developed for the suprachoroidal space that appears less responsive to wound healing^25,26^.

As shown previously by our research group^14^, the stents we used in our study have special physio-chemical antiproliferative properties.

The stents with 75 wt % ZnO-T had elastic modulus values of around 15–20 MPa. This value is higher than that of pure PDMS. The elongation at break is significantly reduced by the addition of 75 wt % of ZnO-T. The stents exhibit ductile behaviour and have an elongation of approximately 30–50% before rupture and are therefore sufficiently stable^14^.

We also examined the potential release of zinc ions in a previous study. For a glass slide sample completely covered with 75 wt% ZnO-T stents, the highest detected amount of dissolved Zn ions in the culture medium was 2.55 ± 0.27 µg/mL after 48 h. Thus, the amount of Zn ions released by a stent is very small and negligible^14^.

As we have shown previously, Zinc Oxide in tetrapodal form have cell-inhibiting properties^13,17^. Due to the particles protruding on the surface, many local contacts with cells occur, which leads to the destruction of the cell membrane. Therefore, Zn ions have the ability to contribute to the cell-inhibiting properties in the environment of the stent^14^.

Though the results of this pilot study are quite promising, we need to note that, similar to the other above mentioned studies^16,19,22^, our number of implanted eyes is small, and that the analyzed time for biocompatibility relatively short. Another limitation is the absence of a control group featuring an implant without ZnO-T. This control group was omitted intentionally, as we previously compared stents made of PDMS only with stents made of PDMS and ZnO-T in vitro and could show significantly fewer cells growing on the PDMS/ZnO-T stent^17^.

In conclusion, this pilot study shows, that functionality and biocompatibility of our newly developed microshunt are highly comparable and in certain aspects even better than reported in similar studies of devices, that are already on the market. Our implant does not cause a negative tissue reaction. Despite these promising results, further pre-clinical studies, such as long-term follow-up for several months in rabbit eyes, further improving the stent’s design and geometry and optimizing the implantation details are planned prior to clinical testing.

## Data Availability

The datasets used and analyzed during the current study are available from the corresponding author on reasonable request.

## References

[1] Sihota R, Angmo D, Ramaswamy D, Dada T (2018). Simplifying, "target" intraocular pressure for different stages of primary open-angle glaucoma and primary angle-closure glaucoma. Indian J. Ophthalmol..

[2] Lamoureux EL (2015). Comparing the effectiveness of selective laser trabeculoplasty with topical medication as initial treatment (the Glaucoma Initial Treatment Study): Study protocol for a randomised controlled trial. Trials.

[3] European Glaucoma Society Terminology and Guidelines for Glaucoma, 5th Edition. *Br. J. Ophthalmol.* **105,** 1–169 (2021). 10.1136/bjophthalmol-2021-egsguidelines 34675001

[4] Chong RS, Crowston JG, Wong TT (2021). Experimental models of glaucoma filtration surgery. Acta Ophthalmol..

[5] Aref AA, Gedde SJ, Budenz DL (2017). Glaucoma drainage implant surgery. Dev. Ophthalmol..

[6] Yu D-Y (2009). The critical role of the conjunctiva in glaucoma filtration surgery. Prog. Retinal Eye Res..

[7] Zhao X, Liu S, Han Y, Wang Y, Lin Q (2021). Preparation of 5-fluorouracil loaded chitosan microtube via in situ precipitation for glaucoma drainage device application: In vitro and in vivo investigation. J. Biomater. Sci. Polym. Ed..

[8] Koz OG (2007). The effect of paclitaxel on conjunctival wound healing: a pilot study. J. Glaucoma.

[9] Takeuchi, K. *et al.* Solid hyaluronic acid film and the prevention of postoperative fibrous scar formation in experimental animal eyes. *Arch Ophthalmol. (Chicago, Ill. : 1960)* **127,** 460–464 (2009). 10.1001/archophthalmol.2009.70 19365025

[10] Ishida K (2021). Evaluation of bleb characteristics after trabeculectomy and glaucoma implant surgery in the rabbit. Ophthal. Res..

[11] van Mechelen RJS (2022). Wound healing response after bleb-forming glaucoma surgery with a SIBS microshunt in rabbits. Translat. Vis. Sci. Technol..

[12] Chatzara A, Chronopoulou I, Theodossiadis G, Theodossiadis P, Chatziralli I (2019). XEN implant for glaucoma treatment: A review of the literature. Semin. Ophthalmol..

[13] Sonntag, S. R. *et al.* Zinc Oxide tetrapods modulate wound healing and cytokine release in vitro-a new antiproliferative substance in glaucoma filtering surgery. *Life (Basel, Switzerland)* **12** (2022). 10.3390/life12111691 PMC9692309 36362846

[14] Gapeeva A (2023). Tetrapodal ZnO-based composite stents for minimally invasive glaucoma surgery. ACS Biomater. Sci. Eng..

[15] Ceresnakova M, Dully M, Murray D, Soulimane T, Hudson SP (2021). Stent conditioned media for in vitro evaluation of hydrophobic stent coatings. Toxicol. Vitro: Int. J. Publ. Assoc. BIBRA.

[16] Fujimoto T (2021). Intraocular pressure-lowering effects of trabeculectomy versus microshunt insertion in rabbit eyes. Translat. Vis. Sci. Technol..

[17] Sonntag, S. R. *et al.* In vitro evaluation of zinc oxide tetrapods as a new material component for glaucoma implants. *Life (Basel, Switzerland)* **12** (2022). 10.3390/life12111805 PMC9697987 36362958

[18] Nakamura K (2022). Tissue reactivity to, and stability of, glaucoma drainage device materials placed under rabbit conjunctiva. Translat. Vis. Sci. Technol..

[19] Acosta, A. C. *et al.* A newly designed glaucoma drainage implant made of poly(styrene-b-isobutylene-b-styrene): Biocompatibility and function in normal rabbit eyes. *Archives of ophthalmology (Chicago, Ill. : 1960)* **124,** 1742–1749 (2006). 10.1001/archopht.124.12.1742 17159034

[20] Lenzhofer M, Hohensinn M, Strohmaier C, Reitsamer HA (2018). Subkonjunktivale minimalinvasive Glaukomchirurgie: Verfahren und klinische Ergebnisse. Der Ophthalmologe : Zeitschrift der Deutschen Ophthalmologischen Gesellschaft.

[21] Fea, A. M. *et al.* Early experience with the New XEN63 implant in primary open-angle glaucoma patients: Clinical outcomes. *J. Clin. Med.* **10** (2021). 10.3390/jcm10081628 PMC8069200 33921311

[22] Shute TS (2016). Biocompatibility of a novel microfistula implant in nonprimate mammals for the surgical treatment of glaucoma. Invest. Ophthalmol. Vis. Sci..

[23] Scheres LMJ (2021). XEN® Gel Stent compared to PRESERFLO™ MicroShunt implantation for primary open-angle glaucoma: two-year results. Acta Ophthalmol..

[24] Lee RMH, Bouremel Y, Eames I, Brocchini S, Khaw PT (2019). The implications of an ab interno versus ab externo surgical approach on outflow resistance of a subconjunctival drainage device for intraocular pressure control. Translat. Vis. Sci. Technol..

[25] García Feijoó, J. *et al.* A European study of the performance and safety of MINIject in patients with medically uncontrolled open-angle glaucoma (STAR-II). *J. Glaucoma* **29,** 864–871 (2020). 10.1097/IJG.0000000000001632 PMC7647427 32769736

[26] Grierson I (2020). A novel suprachoroidal microinvasive glaucoma implant: in vivo biocompatibility and biointegration. BMC Biomed. Eng..

